# The fecal microbiotas of women of Pacific and New Zealand European ethnicities are characterized by distinctive enterotypes that reflect dietary intakes and fecal water content

**DOI:** 10.1080/19490976.2023.2178801

**Published:** 2023-02-17

**Authors:** Nikki Renall, Blair Lawley, Tommi Vatanen, Benedikt Merz, Jeroen Douwes, Marine Corbin, Lisa Te Morenga, Rozanne Kruger, Bernhard H Breier, Gerald W Tannock

**Affiliations:** a School of Sport, Exercise and Nutrition, College of Health, Massey University, Auckland, New Zealand; b Riddet Institute, Centre of Research Excellence, Massey University, Palmerston North, New Zealand; c Research Centre for Hauora and Health, Massey University, Wellington, New Zealand; d Department of Microbiology and Immunology, University of Otago, Dunedin, New Zealand; e Liggins Institute, University of Auckland, Auckland, New Zealand; f Research Program for Clinical and Molecular Metabolism, Faculty of Medicine, University of Helsinki, Helsinki, Finland; g The Broad Institute of MIT and Harvard, Cambridge, MA, USA; h Department of Physiology and Biochemistry of Nutrition, Max Rubner-Institut Karlsruhe, Germany

**Keywords:** Fecal microbiota, enterotypes, dietary intake, fecal water content, obesity

## Abstract

Obesity is a complex, multifactorial condition that is an important risk factor for noncommunicable diseases including cardiovascular disease and type 2 diabetes. While prevention and management require a healthy and energy balanced diet and adequate physical activity, the taxonomic composition and functional attributes of the colonic microbiota may have a supplementary role in the development of obesity. The taxonomic composition and metabolic capacity of the fecal microbiota of 286 women, resident in Auckland New Zealand, was determined by metagenomic analysis. Associations with BMI (obese, nonobese), body fat composition, and ethnicity (Pacific, n = 125; NZ European women [NZE], n = 161) were assessed using regression analyses. The fecal microbiotas were characterized by the presence of three distinctive enterotypes, with enterotype 1 represented in both Pacific and NZE women (39 and 61%, respectively), enterotype 2 mainly in Pacific women (84 and 16%) and enterotype 3 mainly in NZE women (13 and 87%). Enterotype 1 was characterized mainly by the relative abundances of butyrate producing species, *Eubacterium rectale* and *Faecalibacterium prausnitzii*, enterotype 2 by the relative abundances of lactic acid producing species, *Bifidobacterium adolescentis, Bifidobacterium bifidum*, and *Lactobacillus ruminis*, and enterotype 3 by the relative abundances of *Subdoligranulum sp., Akkermansia muciniphila, Ruminococcus bromii*, and *Methanobrevibacter smithii*. Enterotypes were also associated with BMI, visceral fat %, and blood cholesterol. Habitual food group intake was estimated using a 5 day nonconsecutive estimated food record and a 30 day, 220 item semi-quantitative Food Frequency Questionnaire. Higher intake of ‘egg’ and ‘dairy’ products was associated with enterotype 3, whereas ‘non-starchy vegetables’, ‘nuts and seeds’ and ‘plant-based fats’ were positively associated with enterotype 1. In contrast, these same food groups were inversely associated with enterotype 2. Fecal water content, as a proxy for stool consistency/colonic transit time, was associated with microbiota taxonomic composition and gene pools reflective of particular bacterial biochemical pathways. The fecal microbiotas of women of Pacific and New Zealand European ethnicities are characterized by distinctive enterotypes, most likely due to differential dietary intake and fecal consistency/colonic transit time. These parameters need to be considered in future analyses of human fecal microbiotas.

## Background

Obesity (adult body mass index, BMI ≥ 30 kg/m^2^) is a health problem in many countries as it is a risk factor for several non-communicable diseases such as cardiovascular disease and diabetes.^1^ The worldwide prevalence of obesity has tripled since 1975.^1^ Of the OECD countries, adult obesity rates are highest in the USA, Mexico, New Zealand, and Hungary.^2^ Moreover, the prevalence of adult obesity may differ between ethnic groups living in the same country. For example, in New Zealand, 71.3% of Pacific Island, 50.8% of Māori, 31.9% of European/other, and 18.5% of Asian adults are obese.^3^ Obesity is 1.6 times more likely in people in socioeconomically deprived locations in New Zealand.^3^ Clearly, primary disease prevention approaches (optimal nutrition, education, exercise) are required to solve the obesity problem, but ancillary knowledge of biological factors associated with the condition could be important in developing supportive prophylactic measures. One such factor is the gut microbiota that, at least in gnotobiotic animal models, has roles in energy harvest from the digesta, regulation of triglyceride uptake by fat cells and total body fat accumulation.^4^ Obesity in genetically predisposed mice is associated with more bacterial species belonging to the phylum Firmicutes relative to phylum Bacteroidetes, with the opposite observed in lean mice.^5–7^ Partial confirmation of this finding was obtained in a small human study in which the ratio of Firmicutes to Bacteroidetes (F:B) was shown to decrease as obese individuals lost weight while consuming a fat- or carbohydrate-restricted diet.^8^ However, results from murine models have not always been confirmed in human studies, perhaps because the association of the composition of the gut microbiota with obesity is weak, confounded by inter-individual variation and insufficient sample sizes.^9–11^ As concluded by Sze and Schloss,^11^ the involvement of the microbiota in obesity may not be revealed by taxonomic investigations alone but may only be apparent if the metabolic capacity of different gene pools (structures) within the microbial community were investigated. The purpose of our study was to compare the taxonomic composition and metabolic capacity of the fecal microbiota of 286 women who varied principally in BMI (obese, non-obese), body fat composition, ethnicity (Pacific, NZ European), and socioeconomic deprivation level. Comprehensive details of habitual intake of food groups were collected and collated in relation to microbiota data.

## Materials and methods

### Study participants

Two hundred and eighty-six participants were recruited to the cross-sectional study ‘PRedictors linking Obesity and gut MIcrobiomE’ (PROMISE), which was conducted between July 2016 and September 2017. Participants were recruited based on self-reported body mass index (BMI) so that half in each group either had normal BMI or were obese (BMI ≥30 kg/m^2^). Participants were stratified into Body Fat (BF) groups (low-BF% <35%; high-BF% ≥35%) for all subsequent analysis because individuals with the same BMI can have different body compositions and metabolic disease risks.^12–15^ Participants were of Pacific Island origins (n = 125) and NZ European women (n = 161), free from any chronic disease, aged 18–45 years, all resident in the Auckland region. Details of the study procedures and recruitment strategies have been published elsewhere.^16^ Participants made two visits to the research unit and completed questionnaires at home between visits. The study was approved by the Southern Health Disability Ethics Committee (16/STH/32) and conducted according to the guidelines of the declaration of Helsinki. The trial was registered at anzctr.org.au (ACTRN12618000432213). All participants were informed in detail about the procedures and measurements and gave written informed consent.

### Anthropometric and demographic information

Fasting weight and height were used to calculate BMI as kg/m^2^. Body composition was assessed with a whole-body scan using Dual-energy X-ray Absorptiometry (DXA) (Hologic QDR Discovery A, Hologic Inc, Bedford, MA with APEX V. 3.2 software). Total body fat percentage (BF%), and visceral fat percentage were assessed with DXA. The New Zealand Deprivation 2013 index (NZDep2013), an area-based measure of socioeconomic deprivation, was used to assign a socioeconomic deprivation score ranging between decile 1 “least deprived” to decile 10 “most deprived” to each participant.^17^ Blood chemistry assays (glucose, HbA1c, cholesterols, triglycerides, insulin) were conducted using standard diagnostic methods.

### Dietary assessment

Participants completed a 5-day nonconsecutive estimated food record (5DFR) at home. At study visit two, the 5DFR was reviewed in a face-to-face interview with a dietitian and participants completed a validated 220-item semi-quantitative Food Frequency Questionnaire (NZWFFQ) regarding food intake during the previous 30 days.^18^ Energy, macro- and micro-nutrient analyses of the 5DFR and NZWFFQ were completed using FoodWorks9 (Xyris Software [Australia] Pty Ltd, Queensland, Australia) nutrition analysis software, based on New Zealand’s food composition database, FOODFiles 2016 (Plant & Food Research, NZ). All reported energy intakes were reviewed by dietitians and values between 2100 kJ/day and 27000 kJ/day were considered plausible for valid completion of the 5DFR and NZWFFQ; all others (n = 17; Pacific n = 16, NZ European n = 1) were excluded from further analyses. Foods from the 5DFR (n > 2850) and the NZWFFQ were allocated to 55 food groups, based on similar food groupings used in previous studies.^19^ Total energy (reported as kilojoules) includes the energy contribution from all the macronutrients as well as total dietary fiber.

The National Cancer Institute (NCI; USA)^20,21^ method was used to calculate individual usual intakes of the 55 food groups (g/day) consumed for one month for each participant. The NCI method uses a two-part modeling approach to estimate the probability of consumption and the respective amount consumed, considering the effect of covariates which can influence the probability of consumption (e.g., seasonality) or the amount consumed (e.g., age). The individual habitual intake is then defined as the product of probability of consumption multiplied by the consumed amount (Usual intake = Probability x Amount). The 5DFR was used as the primary dietary data, and the covariates age, ethnicity, BMI, season (summer, autumn, winter, spring), weekend (weekday = Monday – Thursday, weekend = Friday – Sunday), and FFQ information (in standard units/day) were included. The 55 food groups were collapsed into 21 food groups, based on similar nutritional composition and characteristics, for all subsequent analyses.

### Fecal microbiota analysis

Fecal samples were collected and stored in the participants’ home freezers 11 to 14 days prior to delivery to the research unit. Subsequent storage was at −80°C until laboratory analysis. Fecal water content, a proxy for colonic transit time, was determined by placing approximately 200 mg of feces in a pre- weighed microfuge tube; the weight was recorded, and the tube with cap open placed in a 37°C incubator. The tubes were subsequently dried until a constant dry weight was obtained, and percentage water content was then calculated.

DNA was extracted from 250 mg feces according to the protocol provided by the manufacturer (PowerSoil DNA isolation kit, Mo Bio, Carlsbad, CA, USA), with the following modification. Fecal samples were suspended in 1 mL of TN150 buffer (containing 10 mM TRIS-CL pH 8.0, 150 mM NaCl). The suspension was centrifuged at 14,600 × g (3 min, 5°C) and the deposit was then resuspended in 700 μl of PowerBead solution from the kit. The suspension was added back to the PowerBead Tubes and the standard protocol followed. DNA was eluted in 100 μl of elution buffer (warmed to 70°C) and then stored at −80°C. Quality and quantity of genomic DNA was checked on a Nanodrop 1000 spectrophotometer (Thermo Fisher Scientific, Waltham, MA, USA) and on a Qubit fluorometer (Thermo Fisher Scientific, Waltham, MA, USA) prior to sending the cleaned DNA to New Zealand Genomics Ltd. (NZGL) for shotgun metagenome sequencing using the Illumina HiSeq 2500 platform (Illumina, San Diego, CA, USA). Libraries were sequenced across a minimum of six HiSeq lanes, and multiple libraries were prepared for several samples to test for library preparation and sequence run bias. An average of 13,150,561 (range 7,6940,894–17,081,755) reads were recovered for each sample. BBDuk (https://sourceforge.net/projects/bbmap/) was used to trim adapters, remove low quality reads and remove reads <100 bp after trimming. KneadData (http://huttenhower.sph.harvard.edu/kneaddata) was used as quality control to remove human genome reads from bacterial reads, implementing the hg19 database. Sequence data from this study is deposited as NCBI Bioproject PRJNA657309.

Microbiota taxonomic profiles were created from DNA sequences using MetaPhlan 2.0 (version 2.6.0) according to default parameters.^22^ Microbiota composition and diversity was further analyzed with QIIME2 (version 2018.8) using converted output tables from MetaPhlan 2.0. Beta diversity group significance for each metric (Bray-Curtis Dissimilarity index, and Jaccard similarity matrix) was measured with PERMANOVA^23^ and group dispersion was measured with PERMDISP.^24^

Enterotypes were predicted in R using the approach described in Arumugum et al.^25,26^ and following the tutorial provided by EMBL (http://enterotyping.embl.de). Differential abundance testing to determine which species were driving enterotypes was carried out with Statistical Analysis of Taxonomic and Functional Profiles (STAMP).^27^ Each enterotype was compared to all other samples using Welch’s *t*-test using Benjamini-Hochberg for multiple testing correction. The association between the species that characterized the enterotypes and fecal water content was explored with Spearman’s correlation.

### Assessment of the metabolic capacity of the microbiota

Metagenomic reads were assembled into contigs using MegaHIT,^28^ individually for each sample, and open reading frames were predicted using Prodigal.^29^ A non-redundant gene catalog was constructed by clustering genes based on sequence similarity at 95% identity and 90% coverage of the shorter sequence using CD-HIT.^30^ Metagenomic gene abundances were estimated by mapping quality trimmed reads from each sample against the gene catalog with k-mer alignment in KMA.^31^ Assembled genes were functionally annotated with eggnog-mapper v2 based on orthology assignments using precomputed eggNOG v5.0 clusters.^32^ Annotations by Gene Onthology (GO) terms, Enzyme Commission (EC) categories, Carbohydrate-Active Enzyme (CAZy) Database identifiers and KEGG Ontology (KO) terms. Data were tested for associations with CAZymes and microbial bile salt hydrolases (BSH) because of previously published associations between these metabolic features of the fecal microbiota and BMI in experimental or clinical situations.^5,33,34^ Associations between metabolic functions and cohort metadata were tested using linear regression, where the abundance of a metabolic function (measured in copies-per-million) was used as the dependent variable and all cohort metadata were used as independent variables simultaneously to control for any confounding.

Metabolic capacity of the microbiota was assessed with HUMAnN2 which quantifies MetaCyc metabolic pathways. Pathway abundances were normalized to copies-per-million units. Associations with subject metadata and MetaCyc pathway abundances were analyzed using linear regression with pathway abundance as a response variable and subject ethnicity, BMI, Shannon index, enterotype, habitual dietary fiber intake (g/day), fecal water content, and F:B ratio as fixed effects.

### Statistical analysis

Analysis of dietary data and associations with microbiota was conducted using SAS Enterprise Guide version 7.1 (SAS Institute, Cary, NC, USA). Normality of data was assessed using Kolmogorov–Smirnov tests and histograms, medians [25th, 75th] were used to present all non-normally distributed continuous data. Mann Whitney tests were used to test for differences between groups. Multiple logistic regression was used to assess associations between habitual food group intake and enterotypes. Univariate analyses were conducted followed by multivariate analyses adjusted for ethnicity, age, deprivation, and energy intake. Analyses were conducted separately for NZE and Pacific participants. Where effect estimates were similar for both groups, analyses for both groups combined were also conducted with analyses adjusted for ethnicity. Further adjustment for BF% group was conducted to assess the independent association of food group intake and enterotypes. The odds of the microbiota reflecting a particular enterotype was expressed per 1 serving size of change in food group intake. Collinearity between variables was assessed by computing the tolerance and the variance inflation factor (VIF); no collinearity was detected. *p*-values <0.05 were considered statistically significant.

## Results

### Anthropometric and demographic information

Complete data sets were obtained for 286 participants: 125 Pacific (44%) and 161 NZE (56%) women. Pacific women were younger (median age 23 y) than NZE women (median age 32 y), had higher BMI and visceral fat values, but lower total and high-density lipoprotein (HDL) cholesterol in comparison to NZE women. NZE women had lower HbA1c, lower fasting plasma concentrations of insulin, and lower Homeostatic Model Assessment of Insulin Resistance (HOMA-IR) scores compared to Pacific women. NZE women resided in less deprived areas than Pacific women. There were no differences in BF% between Pacific and NZE women (Table 1).

**Table 1.** Demographic, metabolic, and body composition data of participants stratified by ethnicity.

	Total population n = 286	Pacific n = 125	NZ European n = 161
Age (years)	28 [22, 35]	23 [20, 29]	32 [25, 37]*
Weight (kg)	77.6 [65.6, 96.0]	82.3 [72.3, 98.8]	70.7 [61.4, 92.5]*
Height (cm)	167.8 [163.7, 172.2]	168.6 [163.8, 174.1]	167.2 [163.7, 171.2]
BMI (kg/m^2^)	28.1 [23.0, 33.4]	29.5 [24.7, 34.8]	25.0 [22.2, 32.9]*
Total body fat (%)	34.5 [28.8, 39.6]	34.6 [29.5, 39.3]	34.3 [27.8, 39.9]
Visceral fat (%)	32.3 [23.7, 38.9]	34.3 [26, 39.8]	30.0 [21.4, 38.3]*
Body fat groups n (%) <35% >35%	152 (53%) 134 (47%)	65 (52%) 60 (48%)	87 (54%) 74 (46%)
Total cholesterol (mmol/L) ∞	4.8 [4.3, 5.4]	4.5 [4.2, 5.1]	5.0 [4.5, 5.8]*
HDL cholesterol (mmol/L) ∞	1.5 [1.3, 1.9]	1.4 [1.3, 1.7]	1.7 [1.4, 2.0]*
LDL cholesterol (mmol/L) ∞	3.0 [2.4, 3.5]	2.9 [2.4, 3.3]	3.1 [2.4, 3.6]*
Triglycerides (mmol/L) ∞	0.9 [0.7, 1.2]	0.9 [0.7, 1.2]	0.9 [0.7, 1.2]
Total cholesterol:HDL ratio (mmol/L) ∞	3.1 [2.6, 3.7]	3.1 [2.6, 3.7]	3.0 [2.5, 3.7]
Glucose (mmol/L) ∞	5.3 [5.0, 5.6]	5.3 [5.1, 5.7]	5.3 [5.0, 5.6]
Insulin (U/ml) ∞	10.9 [7.3, 17.1]	14.6 [9.8, 23.2]	8.7 [6.4, 12.6]*
Homeostatic Model Assessment of Insulin Resistance	2.6 [1.7, 4.0]	3.4 [2.3, 5.9]	2.1 [1.5, 3.1]*
HbA1c (mmol/mol) #	31.7 [30.0, 33.6]	32.9 [31.4, 35.4]	30.7 [29.3, 32.6]*
Deprivation index #	6 [3, 8]	8 [6, 9]	4 [2, 6]*

All values are reported as medians [25th, 75th percentiles] or N (%).

*Statistically significant difference between Pacific and NZ European women (Mann Whitney, p < 0.05).

∞Pacific woman (n = 1) and #Pacific women (n = 2) have not been included in analyses due to missing data.

BMI: body mass index, HDL cholesterol: high density lipoprotein cholesterol, LDL cholesterol: low density lipoprotein cholesterol, HbA1c: glycosylated hemoglobin, Deprivation index: The 2013 New Zealand Deprivation Index.

### Comparison of microbiota compositions

**Figure 1.**
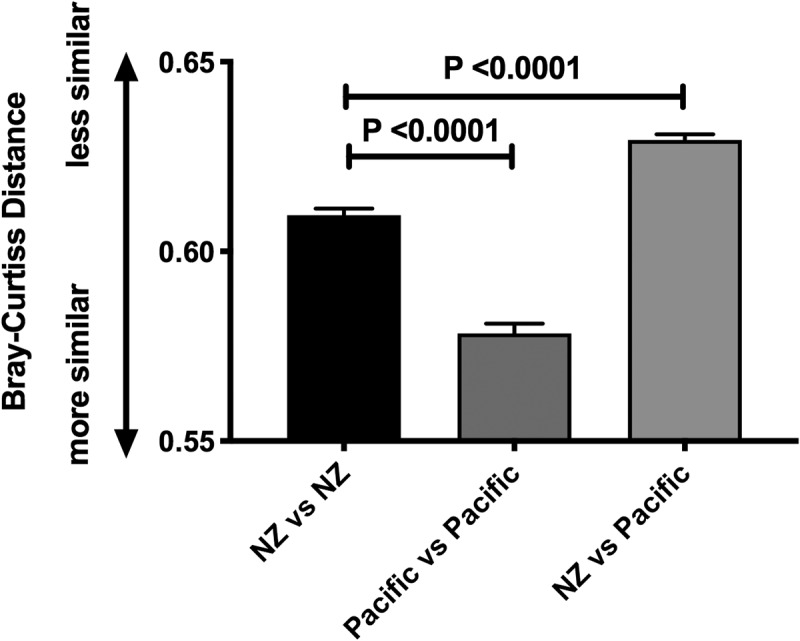
Beta diversity analysis (Bray-Curtis Distance) between fecal microbiotas of NZ European women and Pacific women, within and between ethnic groups.

**Figure 2.**
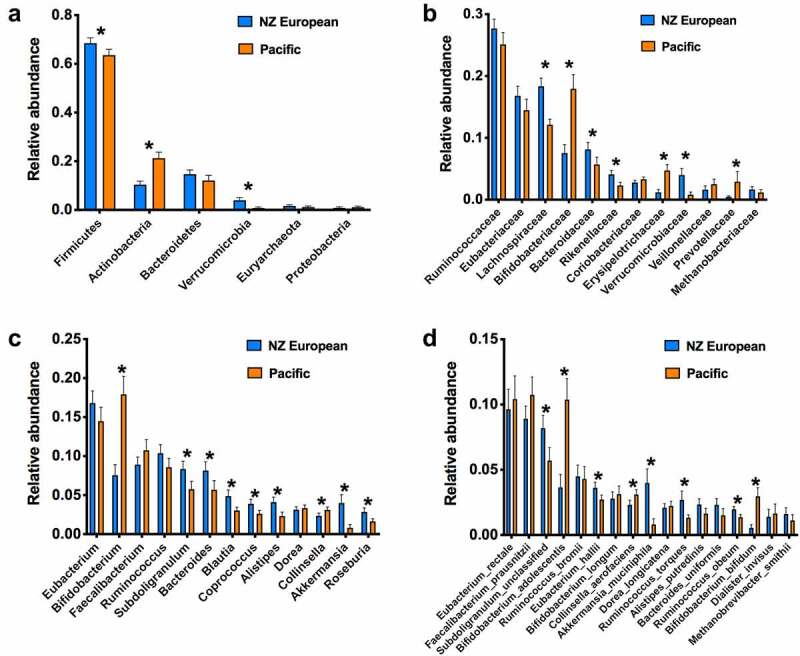
Relative abundances of the most commonly occurring taxa in the feces of NZE and Pacific women (*p<0.05, Benjamini, Krieger, Yekutieli test).

**Figure 3.**
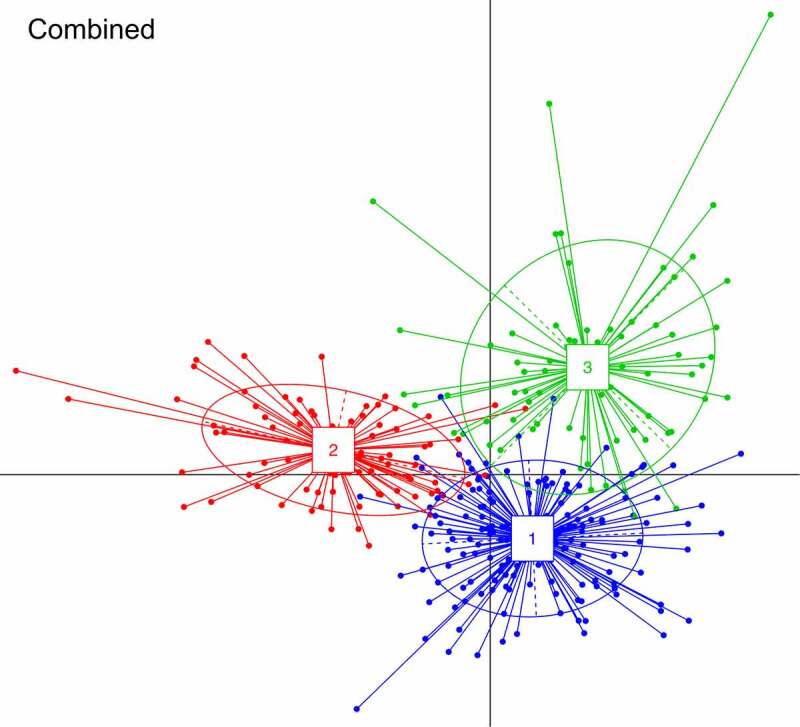
BCA (between-class analysis) plots, showing clustering of samples from combined cohort data into three enterotypes.

**Figure 4.**
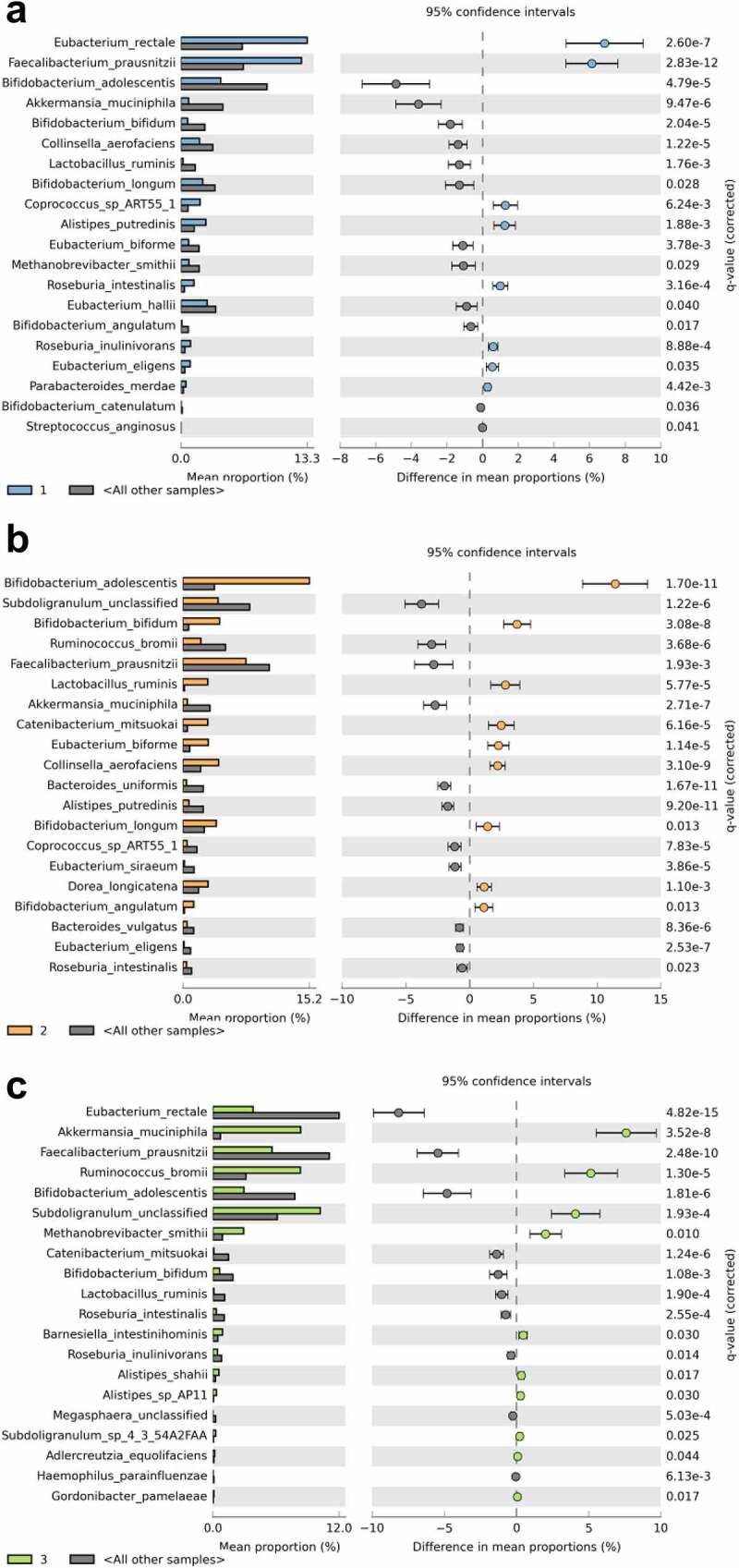
Description of predicted enterotypes. Each panel (a-c) contains the STAMP output (extended error bar plot) depicting species-level features within each enterotype with significant differential abundance (Welch’s *t* test with Benjamini-Hochberg false discovery rate), compared to all other enterotypes.

The median number of predicted species per participant was 73 (Pacific median 75, interquartile range 67–81; NZE 72, 67–77). Stratifying the population by BF% groups did not reveal statistically significant differences in abundances of individual taxa following adjustment for multiple testing (Figure S1). Beta diversity analysis indicated that Pacific and NZE microbiotas were different (Figure 1) and this was confirmed by reference to the relative abundances of taxa present in the feces of the two groups (Figure 2). Searches for robust clusters of bacterial species in the microbiotas detected three enterotypes (Figure 3). Enterotype 1 was characterized mainly by the relative abundances of butyrate-producing species, *Eubacterium rectale* and *Faecalibacterium prausnitzii*. Enterotype 2 was characterized by the relative abundances of lactic acid-producing species, *Bifidobacterium adolescentis, Bifidobacterium bifidum*, and *Lactobacillus ruminis*. Enterotype 3 was characterized by the relative abundances of *Subdoligranulum sp., Akkermansia muciniphila, Ruminococcus bromii*, and *Methanobrevibacter smithii* (Figure 4). Alpha diversity of the fecal microbiota was less in Enterotype 2 individuals relative to those characterized by Enterotypes 1 or 3 (Table 2). Enterotype 1 was found in 146 participants, including both Pacific and NZE women. Enterotype 2 (n = 70) was predominately found in Pacific women, and enterotype 3 (n = 70) predominately in NZE women (Table 2). Women with a microbiota characterized by enterotype 2 were younger and had a higher BMI and visceral fat %, higher fasting insulin, higher HbA1c concentrations and HOMA-IR scores in comparison to women with enterotypes 1 and 3. Women with a microbiota characterized by enterotype 3 were older, had a lower deprivation index, lower HbA1c, lower high density lipoprotein cholesterol ratio (TC:HDL), higher HDL cholesterol, and lower fecal water content compared to those of enterotypes 1 and 2 (Table 2).

**Table 2.** Demographic, metabolic, and body composition data of participants stratified by enterotype.

	Enterotype 1 n = 146	Enterotype 2 n = 70	Enterotype 3 n = 70
Ethnicity n (%) Pacific NZ European	57 (39%) 89 (61%)	59 (84%) 11 (16%)	9 (13%) 61 (87%)
Age (years)	29 [23, 35]^	22 [20, 27]+	31 [24, 38]~
Weight (kg)	75.4 [64.5, 95.0]^	88.3 [68.5, 100.8]+	73.1 [63.9, 90.2]
Height (cm)	168.5 [163.9, 172.7]	168.4 [163.7, 172.6]	166.5 [164.0, 170.2]
BMI (kg/m^2^)	26.3 [22.8, 33.1]^	31.9 [24.6, 35.9]+	25.2 [22.7, 32.4]
Total body fat (%)	34.0 [28.5, 39.3]^	35.5 [31.9, 40.1]	32.5 [27.9, 40.2]
Visceral fat (%)	30.8 [23.1, 37.9]^	35.4 [29.1, 41.3]+	29.1 [20.4, 38.3]
Body fat groups n (%) <35% >35%	83 (57%) 63 (43%)	29 (41%) 41 (59%)	40 (57%) 30 (43%)
Total cholesterol (mmol/L) ∞	4.8 [4.4, 5.5]^	4.6 [4.2, 5.1]	4.8 [4.2, 5.5]
HDL-cholesterol (mmol/L) ∞	1.5 [1.3, 1.9]^	1.4 [1.2, 1.6]+	1.7 [1.4, 2.0]~
LDL – cholesterol (mmol/L) ∞	3.0 [2.5, 3.5]	2.9 [2.5, 3.4]	2.8 [2.4, 3.5]
Triglycerides (mmol/L) ∞	0.9 [0.7, 1.1]^	1.0 [0.8, 1.4]+	0.7 [0.6, 1.2]
Total cholesterol:HDL-cholesterol ratio (mmol/L) ∞	3.1 [2.7, 3.7]^	3.3 [2.7, 4.0]+	2.8 [2.4, 3.3]~
Glucose (mmol/L) ∞	5.3 [5.0, 5.6]^	5.4 [5.1, 5.8]	5.3 [4.9, 5.6]
Insulin (µU/ml) ∞	9.9 [6.9, 14.5]^	16.1 [10.7, 26.9]+	8.7 [6.4, 14.0]
Homeostatic Model Assessment of Insulin Resistance ∞	2.3 [1.5, 3.4]^	3.7 [2.4, 6.3]+	2.2 [1.6, 3.4]
HbA1c (mmol/mol) #	31.6 [29.6, 33.6]^	33.4 [31.7, 35.5]+	30.7 [29.3, 31.9]~
Deprivation index #	5 [3, 8]^	8 [6, 9]+	4 [3, 6]~
Predicted species abundance	72 [64, 78]	73 [67, 78]	76 [69, 81]~
Alpha diversity: Pielou’s Evenness	0.71 [0.66, 0.74]^	0.68 [0.64, 0.71]+	0.70 [0.67, 0.74]
Shannon index	4.4 [4.1, 4.6]^	4.2 [3.9, 4.4]+	4.4 [4.2, 4.6]
Firmicutes:Bacteroidetes ratio	5.2 [2.6, 10.6]^	15.2 [7.0, 28.4]+	5.0 [2.8, 11.3]
Fecal water content (%) ≈	69 [62, 74]	70 [61, 77]+	65 [60, 69]~

All values are reported as medians [25th, 75th percentiles] or N (%).

^Statistically significant difference between enterotype 1 and 2, ~Statistically significant difference between enterotype 1 and 3, +Statistically significant difference between enterotype 2 and 3 (Mann Whitney, p < 0.05).

∞Pacific woman (n = 1), ≈NZE woman (n = 1), and #Pacific women (n = 2) have not been included in analyses due to missing data.

BMI: body mass index, HDL cholesterol: high density lipoprotein cholesterol, LDL cholesterol: low density lipoprotein cholesterol, HbA1c: glycosylated hemoglobin, Deprivation index: The 2013 New Zealand Deprivation Index.

### Firmicutes to Bacteroidetes (F:B) ratio

There were no significant differences in F:B ratio between Pacific and NZE women. However, participants with a low-BF% had a lower F:B ratio (5.2, 95%CL 3.1, 9.5) compared to those with high-BF% (9.8, 95%CL 4.4, 21.3). Enterotype 2 was associated with a greater F:B ratio in comparison to enterotypes 1 and 3. There was no difference in F:B ratio between enterotype 1 and 3 microbiotas (Table 2).

### Fecal water content

There were no significant differences in fecal water content between Pacific and NZE women. However, fecal water content was significantly lower in women with microbiotas reflective of enterotype 3 (Table 2). In addition, the species that characterized enterotype 3 were all inversely correlated with fecal water content: *Akkermansia muciniphila* (r = −0.20, p = 0.001), *Ruminococcus bromii* (r = −0.19, p = 0.001), *Subdoligranulum spp*. (r = −0.23, p < 0.001), and *Methanobrevibacter smithii* (r = −0.14, p = 0.022).

### Association of diet with enterotypes

Enterotypes were associated with habitual intake of particular food groups (Table 3). Participants whose microbiota was characterized by enterotype 1 or enterotype 3 had similar food group intakes but could be differentiated from each other on the basis of intakes of ‘dairy products’, ‘cheese’, ‘non-starchy vegetables’, and ‘egg products’ (Table 3). Participants with microbiotas characterized by enterotype 2, consumed more ‘discretionary savoury foods’ and ‘sugar sweetened beverages’ compared to enterotypes 1 and 3, and less of the food groups that differentiated enterotype 1 from enterotype 3 (Table 3).

**Table 3.** Habitual intake of food groups stratified by enterotype.

Food group g/day	Enterotype 1 n = 146	Enterotype 2 n = 70	Enterotype 3 n = 70
Dairy products	47.4 [21.8, 104.8]	48.0 [31.8, 92.4]~	94.7 [34.4, 159.8]+
Milk alternatives	0.0 [0.0, 30.0]^	0.0 [0.0, 0.0]~	0.0 [0.0, 31.0]
Cheese	13.2 [7.3, 19.7]^	6.8 [5.3, 9.5]~	17.4 [11.5, 24.7]+
Fruit	69.8 [44.5, 107.0]	61.0 [43.4, 106.1]	71.6 [49.4, 125.3]
Non starchy vegetables	83.0 [54.2, 120.8]^	46.0 [36.9, 59.7]~	93.8 [77.7, 126.1]+
Starchy vegetables	77.4 [66.4, 94.6]	73.1 [65.2, 85.1]	74.0 [64.0, 86.8]
Refined breads and cereals	58.5 [46.4, 76.3]^	70.3 [52.4, 84.5]	61.2 [44.1, 84.9]
Unrefined breads and cereals	48.4 [37.0, 66.3]^	40.3 [33.4, 52.8]	46.0 [34.0, 66.6]
Meat and seafood	70.5 [58.5, 81.8]	71.2 [55.3, 88.3]	70.0 [54.9, 81.8]
Processed meat	11.2 [7.4, 16.7]	9.4 [7.5, 14.5]	10.1 [6.8, 12.7]
Eggs	25.3 [18.4, 33.8]^	17.3 [14.7, 23.6]~	31.9 [23.1, 44.4]+
Legumes and meat alternatives	9.6 [5.6, 19.0]^	3.0 [0.0, 6.0]~	11.5 [7.4, 26.5]
Nuts and seeds	3.8 [2.1, 7.8]^	1.9 [0.9, 2.3]~	3.8 [2.2, 9.8]
Animal fat	4.6 [2.9, 7.0]	4.1 [3.0, 6.6]	3.8 [2.7, 7.0]
Plant based fat	9.2 [5.9, 13.9]^	6.1 [4.2, 7.7]~	8.9 [5.6, 13.1]
Savory sauces and condiments	43.7 [25.4, 64.9]^	33.7 [11.4, 47.5]~	54.4 [26.5, 74.2]
Discretionary sweet foods	62.8 [46.7, 88.8]	54.5 [44.1, 87.8]	61.0 [46.6, 84.5]
Discretionary savory foods	98.4 [73.5, 145.0]^	162.2 [119.9, 216.6]~	74.6 [61.0, 99.6]+
Sweetened beverages	251.7 [158.3, 320.9]^	351.1 [271.0, 411.6]~	193.8 [100.5, 250.7]+
Tea, coffee, water	992.4 [531.8, 1527.2]^	779.8 [370.3, 1173.5]~	1197.2 [725.8, 1718.5]
Alcoholic drinks	19.0 [8.0, 35.3]^	10.2 [0.0, 23.8]~	19.6 [11.2, 37.8]

All values are reported as medians [25th, 75th percentiles] Mann Whitney used to identify a significant difference between enterotypes (p < 0.05) ^Statistically significant difference between enterotype 1 and 2 + Statistically significant difference between enterotype 1 and 3 ~ Statistically significant difference between enterotype 2 and 3 Enterotype 1: n = 146 (Pacific n = 57, NZ European n = 89); Enterotype 2: n = 70 (Pacific n = 59, NZ European n = 11); Enterotype 3: n = 70 (Pacific n = 9, NZ European n = 61)

Multiple logistic regression showed significant associations between food groups and enterotype 1 and 3. In particular, for every serving size increase in ‘dairy products’, the likelihood of being characterized as enterotype 1 decreased by 54%, and conversely it increased the likelihood of being characterized as enterotype 3 by 180%. Moreover, for every serving size increase in ‘starchy vegetables’ the likelihood of being characterized as enterotype 1 increased by 145% and it decreased the likelihood of being characterized as enterotype 3 by 68%. Further, a strong positive association was observed between the intake of eggs and enterotype 3. This food group intake was negatively associated with enterotype 2. For every serving size increase in ‘cheese’, ‘non-starchy vegetables’, and ‘nuts and seeds’, the likelihood of being characterized as enterotype 2 decreased by 92%, 68% and 98% respectively (Table 4). Further adjustments for BF% groups did not alter results (data not shown).

**Table 4.** The association of food group intake and the likelihood of being characterized as enterotype 1, 2 or 3.

Food group	Serving size	Enterotype 1^a^ n = 146 OR 95% CI	Enterotype 1^b^ n = 146 OR 95% CI	Enterotype 2^a^ n = 70 OR 95% CI	Enterotype 2^b^ n = 70 OR 95% CI	Enterotype 3^a^ n = 70 OR 95% CI	Enterotype 3^b^ n = 70 OR 95% CI
Dairy products	258 g	**0.46 [0.21,1.00]**	**0.46 [0.21,0.77]**	0.77 [0.36,1.67]	1.00 [0.46,2.80]	**2.80 [1.67,6.05]**	**2.80 [1.29,6.05]**
Milk alternatives	255 g	3.57 [0.77,12.65]	2.77 [0.77,12.65]	**0.01 [0.00,0.22]**	0.02 [0.00,1.00]	1.66 [0.46,5.92]	1.00 [0.22,3.57]
Cheese	40 g	1.68 [0.70,3.96]	1.38 [0.45,4.12]	**0.00 [0.00,0.02]**	**0.08 [0.01,0.92]**	**7.89 [3.02,21.72]**	1.61 [0.50,4.99]
Fruit	150 g	1.00 [0.47,1.82]	0.86 [0.41,1.57]	0.74 [0.30,1.57]	1.82 [0.74,4.45]	1.57 [0.74,2.85]	1.00 [0.47,2.11]
Non starchy vegetables	75 g	**1.82 [1.25,2.84]**	**1.96 [1.16,3.54]**	**0.06 [0.03,0.15]**	**0.32 [0.11,0.86]**	**1.96 [1.25,2.84]**	0.74 [0.44,1.35]
Starchy vegetables	75 g	**2.27 [1.00,5.11]**	**2.45 [1.00,5.50]**	0.59 [0.22,1.45]	0.93 [0.30,2.84]	0.55 [0.22,1.45]	**0.32 [0.11,0.93]**
Refined breads and cereals	40 g	0.79 [0.57,1.08]	0.82 [0.59,1.17]	**1.49 [1.08,2.12]**	1.32 [0.85,1.96]	0.89 [0.59,1.27]	1.08 [0.73,1.55]
Unrefined breads and cereals	40 g	1.04 [0.79,1.32]	1.00 [0.79,1.27]	0.67 [0.41,1.13]	0.85 [0.50,1.49]	1.17 [0.89,1.55]	1.08 [0.82,1.43]
Meat and seafood	65 g	1.07 [0.63,1.79]	1.07 [0.63,1.91]	1.14 [0.63,2.04]	1.07 [0.52,2.04]	0.82 [0.43,1.48]	0.88 [0.40,1.91]
Processed meat	50 g	1.28 [0.35,4.60]	1.35 [0.35,5.32]	0.70 [0.15,3.44]	1.49 [0.23,9.94]	1.00 [0.23,4.38]	0.45 [0.08,2.44]
Eggs	60 g	0.84 [0.34,2.05]	0.66 [0.23,1.82]	**0.02 [0.00,0.10]**	**0.11 [0.00,0.58]**	**14.03 [4.66,43.75]**	**6.62 [1.82,22.17]**
Legumes and meat alternatives	150 g	8.05 [1.00,72.83]	5.16 [0.55,54.40]	**0.00 [0.00,0.00]**	**0.00 [0.00,0.41]**	5.99 [0.86,35.07]	0.74 [0.09,6.94]
Nuts and seeds	30 g	**3.54 [1.35,9.00]**	**3.54 [1.27,9.79]**	**0.00 [0.00,0.01]**	**0.02 [0.00,0.55]**	1.61 [0.70,3.64]	0.56 [0.21,1.52]
Animal fat	20 g	0.90 [0.32,2.60]	0.72 [0.24,2.19]	0.42 [0.10,1.74]	1.00 [0.18,5.50]	2.28 [0.72,6.98]	1.37 [0.41,4.66]
Plant based fat	20 g	**1.91 [1.13,3.21]**	**1.70 [1.00,2.92]**	**0.10 [0.03,0.32]**	**0.21 [0.05,0.83]**	1.17 [0.74,1.88]	0.80 [0.48,1.35]
Savory sauces and condiments	30 g	1.23 [0.97,1.56]	1.20 [0.94,1.56]	**0.51 [0.35,0.72]**	0.76 [0.50,1.16]	1.23 [0.97,1.61]	0.94 [0.70,1.23]
Discretionary sweet foods	40 g	0.96 [0.82,1.17]	1.00 [0.82,1.22]	0.89 [0.70,1.13]	0.89 [0.64,1.22]	1.13 [0.92,1.38]	1.08 [0.89,1.38]
Discretionary savory foods	60 g	0.84 [0.66,1.00]	0.94 [0.70,1.27]	**2.17 [1.71,2.75]**	1.27 [0.89,1.71]	**0.46 [0.32,0.66]**	0.74 [0.48,1.20]
Sweetened beverages	340 g	0.71 [0.36,1.00]	1.00 [0.51,1.97]	**5.45 [2.77,10.72]**	1.40 [0.71,3.89]	**0.26 [0.13,0.51]**	0.71 [0.36,1.97]
Tea, coffee, water	255 g	1.00 [1.00,1.00]	1.00 [1.00,1.00]	**0.77 [0.77,1.00]**	1.00 [0.77,1.00]	**1.00 [1.00,1.29]**	1.00 [1.00,1.29]
Alcoholic drinks	150 g	1.35 [0.86,2.11]	1.16 [0.74,1.82]	0.47 [0.16,1.16]	1.35 [0.64,2.45]	1.00 [0.64,1.57]	0.74 [0.41,1.16]

Total n = 286: Pacific n = 125, NZ European n = 161; Enterotype 1: n = 146 (Pacific n = 57, NZ European n = 89); Enterotype 2: n = 70 (Pacific n = 59, NZ European n = 11); Enterotype 3: n = 70 (Pacific n = 9, NZ European n = 61). ^a^Unadjusted models ^b^Models adjusted for NZDep2013, Age, ethnicity, and energy intake Odds ratio (bolded when statistically significant) represent the change in the outcome per 1 serving size of change in food group intake (presented as the g/serve equivalent e.g., 250 mL of standard cow milk = 258 g). Comparisons focus on the likelihood of being in 1 enterotype compared to both other enterotypes

### Metabolic capacity of the microbiota

**Figure 5.**
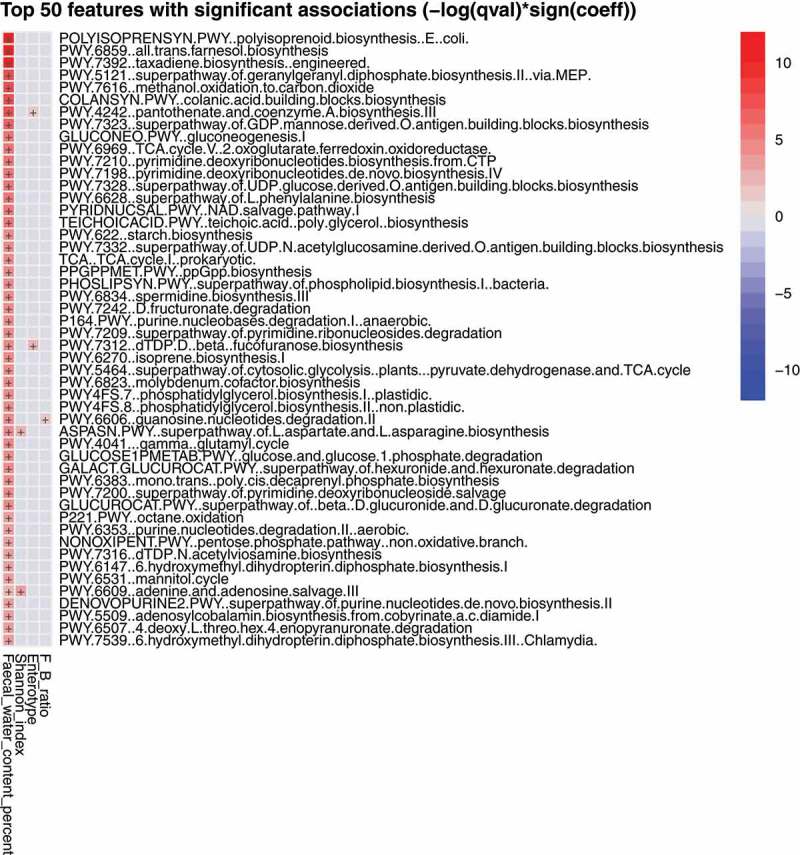
The top 50 most represented biochemical features in the fecal metagenome positively associated with fecal water content. A full list of features is given in Table S1.

The catalog of metagenomically assembled genes contained 3,019,279 non-redundant genes with, on average, 391,362 non-redundant genes per sample (standard deviation 80,183; minimum 128,516; maximum 553,887). We used linear regression to test for associations between EC enzymes, CAZymes, and other participant information, and the specific hypothesis that microbial bile salt hydrolases would be associated with BMI.^33,34^ Associations were not found. However, fecal water content was positively associated with abundance of 117 MetaCyc pathways encoded by the gut microbiota (linear model, FDR corrected p <0.1, top 50 associations summarized in Figure 5; complete data in Table S1). “NAD salvage pathway II”, “all trans farsenol biosynthesis (PWY-6859)”, and “taxadiene biosynthesis (PWY-7392)” were amongst the pathways associated with gut transit time by others.^35^ Shannon index of taxonomic profiles was associated with four MetaCyc pathways (adenine.and.adenosine.salvage.III [PWY.6609], superpathway.of.L.aspartate.and.L.asparagine.biosynthesis [ASPASN.PWY], aminoimidazole.ribonucleotide.biosynthesis.I [PWY.6121], peptidoglycan.biosynthesis.IV.Enterococcus.faecium [PWY.6471]), F:B ratio with two (formaldehyde.assimilation.III.dihydroxyacetone.cycle [P185.PWY], peptidoglycan.biosynthesis.IV.Enterococcus.faecium [PWY.6471]), and enterotype with one (dTDP.D.beta.fucofuranose.biosynthesis [PWY.7312]) (Table S1). Therefore, the main association between specific gene pools was with fecal water content.

## Discussion

The complexity (number of bacterial species per microbiota) was similar between Pacific and NZE groups, but beta diversity analysis indicated differences in microbiota compositions. The microbiotas of the participants could be characterized by the definition of three enterotypes. Thus, enterotype 3 was mostly characteristic of NZE fecal microbiotas, whereas enterotype 2 was mostly characteristic of Pacific microbiotas, which indicated a possible ethnic influence on fecal microbiota composition. Further comparisons revealed that the different categories of microbiotas were also associated with some anthropometric measurements, notably BMI, visceral fat %, and cholesterol values. Thus, enterotyping of fecal microbiotas helped delineate human groups with different ethnic and metabolic characteristics. The enterotypes that we describe here are different from those previously described in relation to stratification of microbiota composition.^25^ Commonly, three enterotypes have been observed, usually characterized by the abundance of the genera *Bacteroides* (E1), *Prevotella* (E2), or *Ruminococcus* (E3). We used the same bioinformatics approach for detection of enterotypes as described by others^25^ based on the Calinski-Harabasz index, but our taxonomic data is derived from MetaPhlAn analysis of shot-gun DNA sequencing which provides accurate species discrimination. Together with STAMP, we find that robust clusters of bacterial species whose relative abundances differ between individuals characterize and differentiate microbiotas.

The composition of the microbiota could be confounded by features of human diet, environment, and innate physiology.^36–38^ Indeed, we found that fecal water content was different between subjects whose microbiotas belonged to enterotype 3 compared to enterotypes 1 and 2. Fecal water content was used as a surrogate measurement of stool consistency which has been considered to reflect gut transit time.^37,39^ The Bristol Stool Form Scale is often used as a tool for subjective scoring of stool consistency but has only moderate correlation when used by untrained participants.^40^ Fecal dry weight (fecal water content) has been recommended as a reliable method to assess stool consistency because it is an objective measurement and has high reproducibility.^40^ Fecal water content is also influenced by the water-holding capacity of insoluble solids, and the water-absorbing ability of the participant.^41^ Enterotype 3 participants had feces of lower water content, indicating slower colonic transit time. This enterotype was characterized by the relative abundances of *Subdoligranulum* sp., *Akkermansia muciniphila, Ruminococcus bromii*, and *Methanobacter smithii* and is consistent with the observations of Vandeputte and colleagues.^42^ They found an increased abundance of methane-producing bacteria (*Methanobrevibacter), Akkermansia*, and *Ruminococaceae* in association with stool consistency indicative of slower gut transit time. An association of Archaea with firmer stool consistency was also reported by Tigchelaar et al.^43^ Both these studies used Bristol Stool Scale assessment of fecal properties. Gut transit time can be measured accurately as the duration of time from ingestion of blue dye within a standardized food to its first excretion of blue color within a stool. Asnicar and colleagues^35^ used this method to measure gut transit time and reported that longer transit time was associated with increased abundances of *Akkermansia muciniphila, Bacteroides*, and *Alistipes* spp. Steenackers et al.^44^ linked alterations in fecal microbiota composition specifically to colonic transit time. This topic has been reviewed recently by Prochazkova and colleagues^45^ who concluded that disease-related microbiota compositions may be confounded by changes in gut transit time. There is, therefore, considerable agreement between studies on the influence of colonic transit time on the taxonomic composition of fecal microbiotas. As in our study, functional pathways and gene families represented in fecal metagenomes were also assessed by Asnicar et al.^35^ “NAD salvage pathway II”, “all trans farsenol biosynthesis (PWY-6859)”, and “taxadiene biosynthesis (PWY-7392)” were amongst the pathways that had greater representation in fecal metagenomes associated with slower gut transit. We also detected different abundances of genes associated with these pathways, but there was a positive rather than a negative correlation with water content of feces. Overall, knowledge of metabolic capacity of microbiotas did not aid in the discrimination of groups with different BMI or body fat composition.

*Bifidobacterium* abundance has been inversely correlated with the fat content of the diet rather than stool consistency/gut transit time.^39^ Higher bifidobacterial abundances characterized enterotype 2 and significant negative associations between the intake of the food groups ‘plant-based fat’ and ‘nuts and seeds’, which are characteristically high in unsaturated fatty acids, were observed.

Limitations of our study include the use of self-reported dietary data that may be prone to misreporting but which nevertheless provide insight into eating habits unavailable by other means.^46^ Lifestyle factors other than diet (for example, amount and type of physical activity) could influence metabolic health profiles and are under investigation with the study participants.^47^

The results of our study pointed to marked associations with ethnicity on microbiota composition because 84% of Pacific women had microbiotas characterized by enterotype 2. Of particular note, the Firmicutes to Bacteroidetes (F:B) ratio was greater in participants with enterotype 2 microbiotas. As reported by others, higher F:B ratio was associated with higher BMI values. Ethnic differences in microbiota composition point to the potential impact of human genetics, and/or fecal microbiotas of family members, and/or general household environment (including habitually ingested foods) on the composition of the gut microbiota.^26,48^ Comparison of ethnicities in previous studies sometimes shows differences in fecal microbiota composition.^49–53^ However, the differences are likely be due to lifestyle differences, and environmental factors that tend to be characteristic of ethnic groups including dietary patterns, and living and working in similar neighborhoods, rather than ethnicity per se.^26,48,54^ All things considered, the genotype of the host probably has little impact on the composition of the adult microbiota which is mainly influenced by environmental factors such as diet and shared environment.^55^ Indeed, we observed significant associations between food group intake and enterotypes. Higher intakes of egg and dairy products increased the likelihood of being characterized as enterotype 3, which primarily consisted of older NZE women. Moreover, ‘non-starchy vegetables’, ‘nuts and seeds’ and ‘plant-based fats’ were positively associated with the likelihood of being characterized as enterotype 1, detected in both Pacific and NZE microbiotas. These food groups were inversely associated with enterotype 2, primarily detected in Pacific women. It is unlikely that the differences we observed were driven by study design (i.e., selectively recruiting low- and high-BF% groups) because the observations were independent of further adjustment for BF% groups. Clearly, the causative role of specific food groups identified in our study on the selection of enterotypes detected in feces needs to be further investigated through dietary intervention studies.^56^

## Conclusions

Sze and Schloss^11^ proposed that evaluation of metabolic capacity represented by different gene pools within the microbiota might assist in understanding the contribution of the microbial community to obesity. Although we observed differences in the representation of biochemical pathways among participants, this was associated with fecal water content (stool consistency/gut transit time) rather than BMI or body fat percentage. Importantly, our study of NZ women showed differences in the taxonomic compositions and metabolic capacities of the fecal microbiota that were associated with dietary intakes and fecal water content. Clearly, future studies in which fecal microbiotas are characterized must be conducted with cognizance of the ethnicity, habitual dietary intake, and colonic transit times of the participants.

## Supplementary Material

Supplemental Material
