# Supplementary figures and images for: The fecal microbiotas of women of Pacific and New Zealand European ethnicities are characterized by distinctive enterotypes that reflect dietary intakes and fecal water content
Source: Gut Microbes. 2023 Feb 17;15(1):2178801. doi: 10.1080/19490976.2023.2178801 (PMC9980675; doi:10.1080/19490976.2023.2178801)

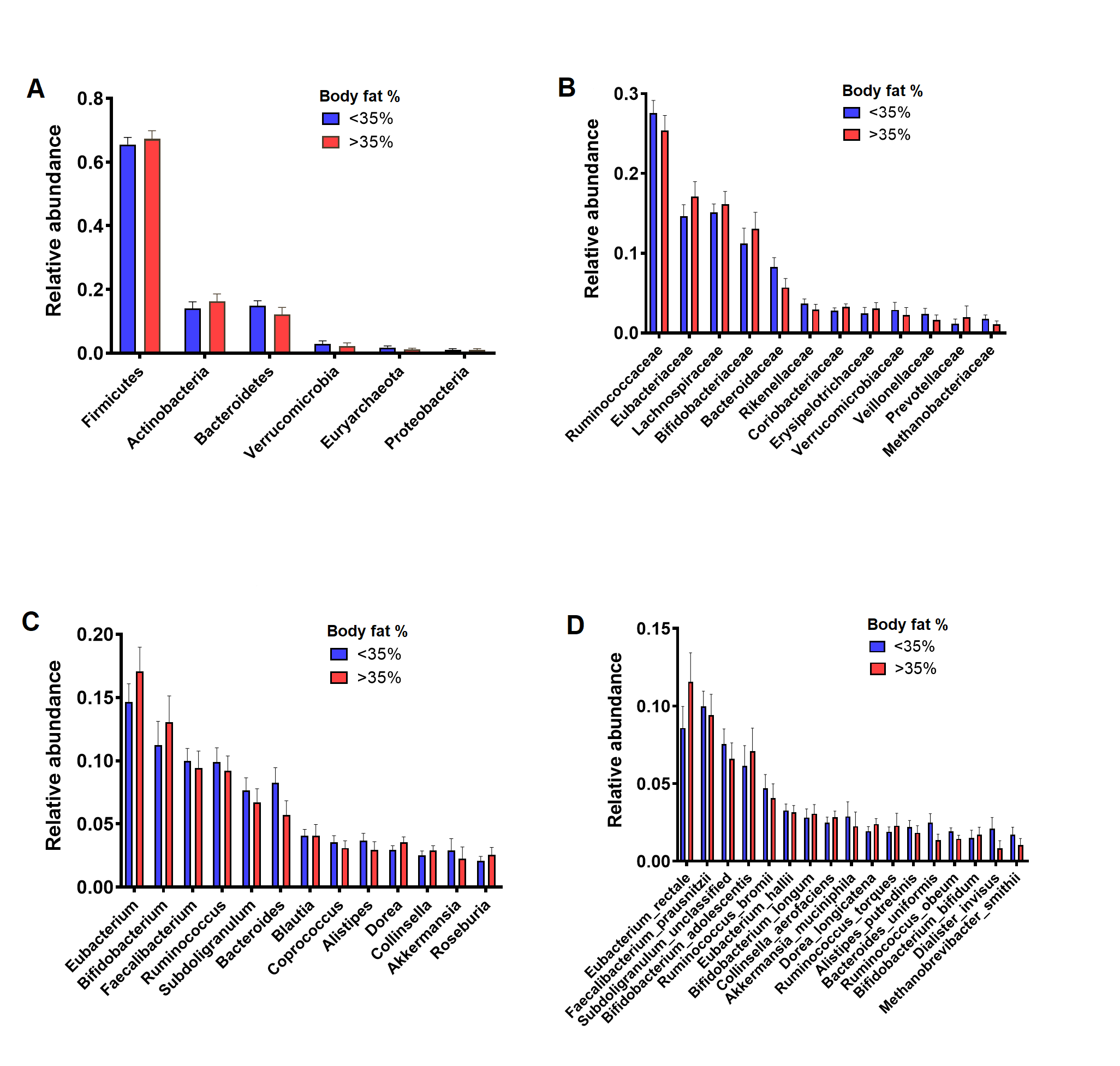

Supplement: Supplemental Material [file KGMI_A_2178801_SM5275.zip › FIGURE S1.tif]
